# One-Step Carbon Coating and Polyacrylamide Functionalization of Fe_3_O_4_ Nanoparticles for Enhancing Magnetic Adsorptive-Remediation of Heavy Metals

**DOI:** 10.3390/molecules22122074

**Published:** 2017-11-27

**Authors:** Mohamed A. Habila, Zeid A. ALOthman, Ahmed Mohamed El-Toni, Joselito Puzon Labis, Aslam Khan, Adel Al-Marghany, Hussein Elsayed Elafifi

**Affiliations:** 1 Chemistry Department, College of Science, King Saud University, Riyadh 11451, Saudi Arabia; zaothman@ksu.edu.sa (Z.A.A.); amarghany@KSU.EDU.SA (A.A.-M.); 2 King Abdullah Institute for Nanotechnology, King Saud University, Riyadh 11451, Saudi Arabia; jlabis@ksu.edu.sa (J.P.L.); aslamkhan@ksu.edu.sa (A.K.); helafifi@ksu.edu.sa (H.E.E.); 3 Central Metallurgical Research and Development Institute, CMRDI, Helwan, 11421 Cairo, Egypt

**Keywords:** adsorption, water treatment, magnetic separation, nanotechnology, solvothermal technique, heavy metals

## Abstract

Magnetic nanoparticles are used in adsorptive removal of heavy metals from polluted wastewater. However, their poor stability in an acidic medium necessitates their protection with a coating layer. Coating magnetic nanoparticles with carbon showed proper protection but the heavy metal removal efficiency was slightly weak. However, to boost the removal efficiencies of surface functionalization, polyacrylamide was applied to carbon-coated Fe_3_O_4_ nanoparticles. In this paper, to facilitate the synthesis process, one-step carbon coating and polyacrylamide functionalization were conducted using the hydrothermal technique with the aim of enhancing the adsorptive removal capacity of Fe_3_O_4_ nanoparticles towards some heavy metals such as Cu(II), Ni(II), Co(II), and Cd(II). The results showed that the one-step process succeeded in developing a carbon coating layer and polyacrylamide functionality on Fe_3_O_4_ nanoparticles. The stability of the magnetic Fe_3_O_4_ nanoparticles as an adsorbent in an acidic medium was improved due to its resistance to the dissolution that was gained during carbon coating and surface functionalization with polyacrylamide. The adsorptive removal process was investigated in relation to various parameters such as pH, time of contact, metal ion concentrations, adsorbent dose, and temperature. The polyacrylamide functionalized Fe_3_O_4_ showed an improvement in the adsorption capacity as compared with the unfunctionalized one. The conditions for superior adsorption were obtained at pH 6; time of contact, 90 min; metal solution concentration, 200 mg/L; adsorbent dose, 0.3 g/L. The modeling of the adsorption data was found to be consistent with the pseudo-second-order kinetic model, which suggests a fast adsorption process. However, the equilibrium data modeling was consistent with both the Langmuir and Freundlich isotherms. Furthermore, the thermodynamic parameters of the adsorptive removal process, including Δ*G*°, Δ*H*°, and Δ*S*°, indicated a spontaneous and endothermic sorption process. The developed adsorbent can be utilized further for industrial-based applications.

## 1. Introduction

Water contamination with heavy metals is a source of concern since the metal cations tend to accumulate within the environment [1]. Rain and snow can wash toxic metal elements into lakes, reservoirs, and underground water [2]. Cadmium, nickel, copper, cobalt, etc. exist in industrial wastewater and upon transfer to water streams can lead to bioaccumulation in living systems that can cause health issues in animals and humans such as cancer or kidney failure [3,4]. Therefore, improvement of the adsorbents for higher adsorption ability for removal of metal cation pollution from water is required and considered a priority research topic. The traditional methods for metal pollution remediation include chemical precipitation, electro-flotation, ion exchange, reverse osmosis, and adsorption, which is the recommended method [5,6,7,8,9,10,11,12,13]. Nanostructure adsorbents have exhibited much higher efficiency and faster adsorption rates in water treatment when compared to traditional ones [14,15]. However, the main drawbacks of nanostructure adsorbents are the difficulty of their separation. Magnetic nanomaterials can be easily and rapidly separated from an aqueous solution under an external magnetic field due to their features of simplicity, efficiency, and sensitivity [16,17,18,19,20,21].

The enhancement of the adsorptive removal capacity of magnetic nanoparticles can be done either by surface functionalization or by improving the chemical stability [22,23,24,25,26,27]. Usually, adsorption of heavy metal cations takes place in an acidic medium, while Fe_3_O_4_ is not stable in this condition. Therefore, it is necessary to protect Fe_3_O_4_ nanoparticles by surface coating to make them stable in this environment. Carbon coating was applied to protect magnetic nanoparticles within an acidic medium and the carbon layer was also utilized for adsorptive removal of heavy metals. Additionally, a protective coating layer was implemented together with attaching some functional groups to improve their adsorption efficiency [28]. Tang et al. (2012) synthesized Fe_3_O_4_@mesoporous silica composite microspheres (MS) and polyethylenimine(PEI) functionalization of Fe_3_O_4_@mesoporous silica composite microspheres by solvothermal reaction for humic acid adsorption [29], where the silica shell acted as a protective coating and PEI was the surface functional moiety. Tang et al. (2013) prepared amino-functionalized Fe_3_O_4_@mesoporous SiO_2_ core-shell composite microspheres for Pb(II) and Cd(II) adsorption and silica worked as a protective layer with immobilized amino groups to chelate heavy metal cations [30]. However, most of these reports implemented two-step synthesis processes where in the first step was to develop a protective coating layer and the second was to impart a functional group. Therefore, this work aims to facilitate the synthesis process through developing one-step carbon coating and polyacrylamide functionalization using a hydrothermal technique to enhance the adsorptive removal capacity of Fe_3_O_4_ nanoparticles towards some heavy metals, such as Cu(II), Ni(II), Co(II), and Cd(II). Factors controlling the adsorption of these heavy metals such as pH, time of contact, and concentration of metal ion solutions were optimized. Different kinetic models, isotherms, and thermodynamic equations were applied to the adsorption data.

## 2. Results and Discussion

### 2.1. Properties of the Fabricated Polyacrylamide Functionalized Magnetic Nanoparticles

**Figure 1:**
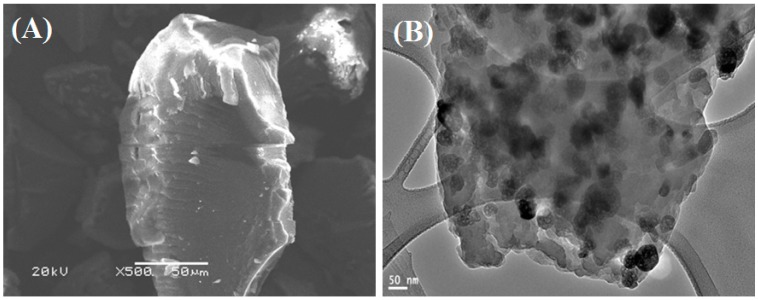
(**A**) SEM and (**B**) TEM of polyacrylamide functionalized Fe_3_O_4_ nanoparticles.

The application of the adsorption process for heavy metal removal depends mainly on the surface functional groups of the adsorbent and their morphology. Therefore, the fabricated polyacrylamide-functionalized magnetic nanoparticles were characterized by SEM, TEM, XRD, and FTIR. The SEM image showed the rough surface morphology of the particle which contains some cavities (Figure 1A). TEM image (Figure 1B) showed that the magnetic nanoparticles were uniform, with an average particle size of around 50–70 nm. The magnetic nanoparticles were impeded within the carbon matrix formed by carbonization of glucose molecules under the hydrothermal condition. Moreover, the magnetic Fe_3_O_4_ nanoparticles were evenly and homogenously distributed within the carbon matrix.

**Figure 2:**
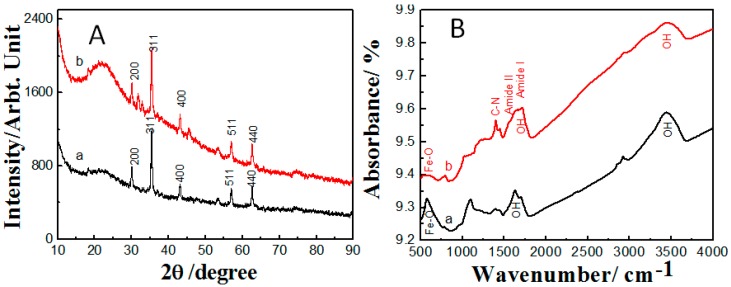
(**A**) XRD patterns and (**B**) FTIR spectra of (a) bare Fe_3_O_4_ and (b) polyacrylamide functionalized Fe_3_O_4_ nanoparticles.

To investigate the crystalline character of Fe_3_O_4_ nanoparticles before and after surface functionalization, X-ray diffraction spectra for bare and polyacrylamide functionalized Fe_3_O_4_ nanoparticles were conducted, as shown in Figure 2A. The X-ray diffraction pattern for the bare Fe_3_O_4_ showed the existence of six peaks that matched well with the pure magnetite phase ((220), (311), (400), (511), and (440) planes of fcc structured Fe_3_O_4_ (JCPDS, 85-1436)), which suggests the presence of a cubic crystal system [31]. Upon performing surface functionalization using polyacrylamide, the X-ray diffraction pattern did not show any noticeable changes at the peaks, which suggests the maintaining of the Fe_3_O_4_ cubic phase. However, there was a slight reduction in the peak intensity that can be attributed to the shielding effect of the polyacrylamide matrix [32,33]. In order to examine the surface functionality of the synthesized polyacrylamide functionalized Fe_3_O_4_ nanoparticles, FTTR measurements were carried out for the bare and polyacrylamide functionalized Fe_3_O_4_ nanoparticles, displayed in Figure 2B. The FTIR spectra of the bare Fe_3_O_4_ sample showed a peak at 585 cm^−^, which is due to Fe–O bonding vibration, while the peak appearing at 3420 cm^−1^ arose from OH^−^ adsorbed on the Fe_3_O_4_ nanoparticles. However, after functionalization of Fe_3_O_4_ with polyacrylamide, the peak characteristic for Fe–O bonding at 570 cm^−1^ was suppressed, which can be attributed to the shielding effect of the polyacrylamide layer [32,33]. The main functional group of polyacrylamide (amide I and amide II) appeared as a broad band (1580–1700 cm^−1^), which also interfered with the OH functional group that existed in the same range. The amide II group that refers to the bending vibration of NH_2_ was noticed at 1620 cm^−1^, while the amide I group that refers to C=O bonding existed at 1680 cm^−1^. Moreover, the third functional group of polyacrylamide was observed at 1430 cm^−1^ for stretching vibration of C–N bonding. Additionally, a broad peak from 3300–3600 cm^−1^ was observed, which is related to the combination of the peaks of NH and OH. 

To evaluate the stability of Fe_3_O_4_ in an acidic medium after functionalization with polyacrylamide, bare and polyacrylamide functionalized Fe_3_O_4_ nanoparticles were soaked in 0.1 M HCl and the results are shown in Figure S1 (see Supplementary Materials). The results represent the dissolution percentage in the case of bare Fe_3_O_4_, which was considered to be 100% compared with 20% in the case of polyacrylamide fununctionalized Fe_3_O_4_. This means that polyacrylamide functionalized Fe_3_O_4_ was stable and protected from dissolution in an acidic medium, making it more proper for heavy metal adsorption, which usually occurs in an acidic medium. 

**Figure 3:**
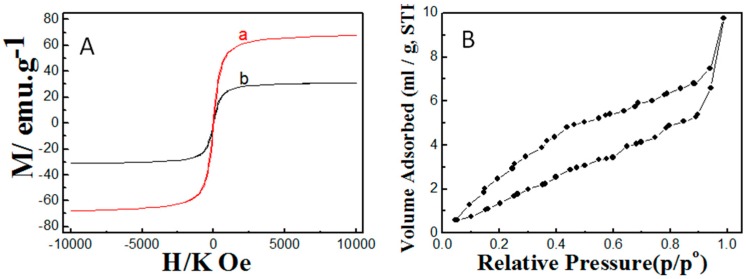
(**A**) Magnetic strength of (a) bare Fe_3_O_4_ and (b) polyacrylamide functionalized Fe_3_O_4_; (**B**) nitrogen sorption of polyacrylamide functionalized Fe_3_O_4_ nanoparticles.

To elucidate the effect of the carbon coating as well as polyacrylamide functionalization on the magnetic character of Fe_3_O_4_, the magnetic property was measured for the bare Fe_3_O_4_ and polyacrylamide-functionalized Fe_3_O_4_ as shown in Figure 3A. It is clear that Fe_3_O_4_ showed high magnetic strength with a saturated magnetization of 67 emu·g^−1^. After performing carbon coating and polyacrylamide functionalization, there was a noticeable reduction in the magnetic strength to reach saturated magnetization of 31 emu·g^−1^. However, after such reduction in magnetic strength, polyacrylamide-functionalized Fe_3_O_4_ can still be easily separated from the solution under the effect of the external magnetic field. The textural properties, specific surface area, and total pore volume were also evaluated for polyacrylamide-functionalized Fe_3_O_4_ through performing the N_2_ sorption isotherm, which is displayed in Figure 3B. It can be seen that the isotherm possessed type IV, which may be due to the non-ordered pores and cavities within the polyacrylamide-functionalized carbon-coated Fe_3_O_4_ matrix. The specific surface area of 8.225 m^2^/g and total pore volume of 0.015 cc/g are indicative of weak textural properties and suggest their limited contribution to the adsorptive removal of heavy metal cations.

### 2.2. Adsorption Characteristics

#### 2.2.1. Effect of pH and Concentration of Heavy Metal Solution

**Figure 4:**
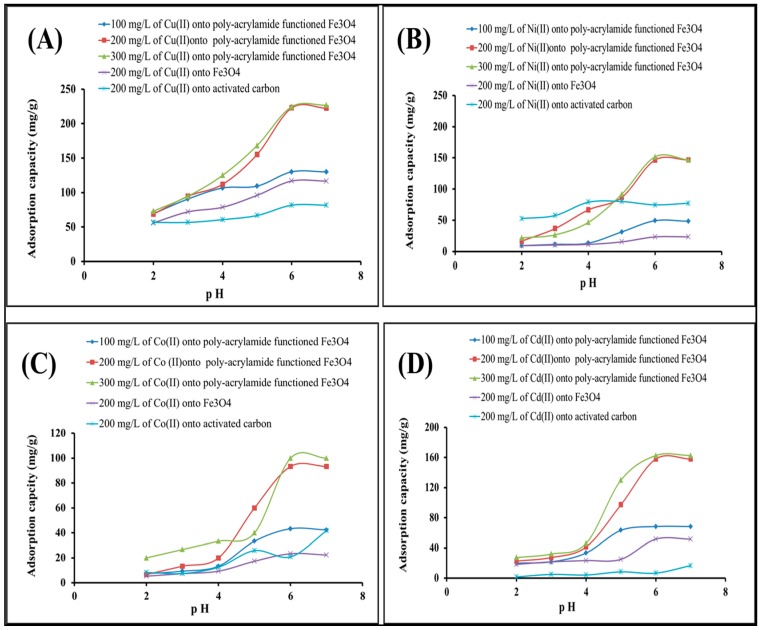
Effect of pH of (**A**) Cu(II); (**B**) Ni(II); (**C**) Co(II); and (**D**) Cd(II) on adsorption capacity upon mixing 0.5 g/L activated carbon with 200 mg/L of a metal cation solution, 0.5 g/L Fe_3_O_4_ nanoparticles, and 0.5 g/L polyacrylamide functionalized Fe_3_O_4_ nanoparticles with 100, 200, and 300 mg/L solutions of metal cations and 25 °C.

The adsorption process is significantly affected by the pH of metal cations in an aqueous solution. Controlling the pH or concentration of hydrogen ions could replace the metal cations on the active sites of adsorbent surface and finally impact the ionization behavior of metal cations during the adsorption process [25]. The effects of pH on heavy metal cations adsorption at an initial concentration of 100, 200, and 300 mg/L for the abovementioned metal cations are shown in Figure 4. Additionally, Fe_3_O_4_ is applied as an adsorbent at a metal concentration of 200 mg/L, where the obtained adsorption capacity was quite low compared with polyacrylamide modified Fe_3_O_4_ (Figure 4). At low pH values (>4), the adsorption capacities of Cu(II), Ni(II), Co(II), and Cd(II) on polyacrylamide functionalized Fe_3_O_4_ were low, while at higher pH values (4 to 7) the adsorption capacities showed much higher values. However, the maximum adsorption capacity was obtained at a pH of 6. The acidic medium is reported to allow high efficiency for heavy metals’ adsorptive removal due to the competition between H^+^ and heavy metals for the binding sites of polyacrylamide functionalized Fe_3_O_4_, resulting in a decrease in their adsorption capacity in the strongly acidic medium [25]. Furthermore, amide groups in the polyacrylamide-functionalized magnetic nanoparticles are protonized at a low pH, which results in the passivation of adsorption sites and hence reduces the metal adsorption capacities. These results are in agreement with the findings of Ayub et al. and Hashem et al., who reported that heavy metal cations are completely released from adsorption sites under strongly acid conditions [34,35]. Upon increasing the concentration of heavy metal from 100 mg/L to 200 mg/L, it can be noticed that the adsorption capacity has been promoted for heavy metal cations. However, there is no significant change of adsorption behavior when elevating the concentration of heavy metal from 200 mg/L to 300 mg/L. For comparison purposes, the activated carbon adsorption capacity was tested for the removal of heavy metal cations of 200 mg/L concentrations at the same conditions. The results shown in Figure 4 indicate that activated carbon without any modification has a poor adsorption capacity for heavy metals as compared to the polyacrylamide functionalized Fe_3_O_4_.

#### 2.2.2. Kinetics of Adsorption

**Figure 5:**
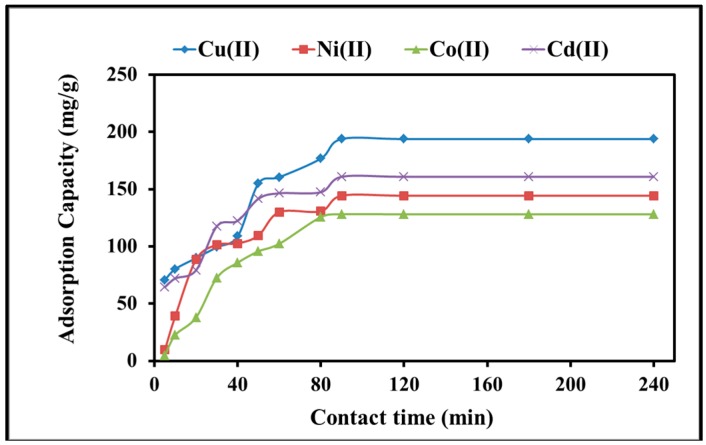
Effect of time of contact of heavy metal cations on the adsorption capacity upon mixing 0.3 g/L polyacrylamide functionalized Fe_3_O_4_ nanoparticles with 150 mg/L metal cations solution at pH 6 and 25 °C.

The rate of the adsorption process was predicted by examining kinetic parameters. In order to elucidate adsorption process kinetics, the time needed to attain the equilibrium as well as the highest heavy metal adsorption should be addressed. The change in concentration of heavy metals in the adsorption mixture was observed by measuring the decrease in the heavy metal concentration overtime. In this regard, the time of contact of polyacrylamide functionalized magnetic nanoparticles with a solution containing heavy metals varied in the range of 10–120 min. The time of contact optimization results are displayed in Figure 5. It can be seen that heavy metals were rapidly adsorbed onto the surface of polyacrylamide functionalized Fe_3_O_4_ within 90 min. However, contact was continued for up to four hours to confirm the achievement of equilibrium status. The adsorption behavior differs from one element to another; the Cu cations showed the maximum adsorption capacity, while Co showed much less. The time of contact results suggest that 90 min was adequate to accomplish equilibrium. From Figure 5, the adsorption capacity did not change effectively with further increments in the time of contact. At 90 min, the adsorption capacity was recorded at 194, 144.3, 128, and 161 mg/g for Cu(II), Ni(II), Co(II), and Cd(II), respectively, which are considered the maximum adsorption capacities.

The pseudo-first-order equation of Lagergren, as described by Wen et al. was applied to the adsorption of heavy metal cations onto 0.3 g/L polyacrylamide functionalized Fe_3_O_4_ nanoparticles at pH 6, and 25 °C. The equation is generally expressed in the integrated form of Equation (1) [36]:

log(*q_e_* − *q_t_*) = log*q_e_* − *k*_1_*t*/2.303 (1)

**Figure 6:**
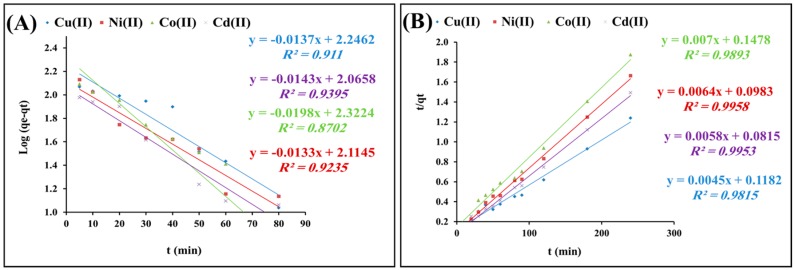
Pseudo-first-order (**A**) and pseudo-second-order; (**B**) of heavy metal adsorption tests upon mixing 0.3 g/L polyacrylamide functionalized Fe_3_O_4_ nanoparticles with 150 mg/L metal cations solution at pH 6 and 25 °C.

The pseudo-first-order rate constant, *k*_1_, was calculated from the slope of the graph of log (*q_e_* − *q*) versus time *t* (Figure 6A). The calculated *k*_1_ values and corresponding linear regression correlation coefficient values are shown in Table 1. It is clear that the linear regression correlation coefficient value R_1_^2^ was no more than 0.93 and the calculated *q_e_* values (239.8, 128.8, 208.9, and 114.8 mg/g) were significantly different from the experimental *q_e_* ones (194, 144.3, 128, and 161 mg/g) for Cu(II), Ni(II), Co(II), and Cd(II), respectively. The results indicate that the pseudo-first-order model cannot be applied to predict the adsorption kinetics.

In addition, the pseudo-second-order kinetic rate equation was applied to the obtained adsorption capacities to evaluate their fittingness. The equation is expressed in the integrated form of Equation (2) [37]:

*t*/*q_t_* = 1/*Kq_e_*^2^ + 1/*q_e_t* (2)

where *t* refers to the time of contact (min), and *q_e_* (mg/g) and *q_e_*^2^ (mg/g) refer to the amount of solute adsorbed at equilibrium for adsorption of heavy metal cations onto 0.3 g/L polyacrylamide-functionalized Fe_3_O_4_ nanoparticles at pH 6 and 25 °C. Figure 6B shows the linear relationship of the graph plot of *t*/*q_t_* versus *t*, from which *q_e_* and *k* can be determined from the slope and intercept, respectively. 

The calculated *k*_2_ values and corresponding linear regression correlation coefficient values are shown in Table 2. Results showed that the calculated *q_e_* values (200.18, 150.7, 130.6, and 167.7 mg/g) more closely fit the experimental *q_e_* data (194, 144.3, 128, and 161 mg/g) for Cu(II), Ni(II), Co(II), and Cd(II), respectively. Moreover, the model fitting was much better than for the pseudo-first-order one. According to these results, it can be said that the pseudo-second-order kinetic model provided a good correlation for the description of the mechanism of sorption of heavy metals onto polyacrylamide functionalized Fe_3_O_4_ nanoparticles. From these results, it can be concluded that the adsorption process is fast and the rate of the reaction is mainly controlled by migration of the metal ions from solution to the surface of the adsorbent and migration of the metal ions to the pores of the adsorbent [38,39].

#### 2.2.3. Effect of Adsorbent Dose

**Figure 7:**
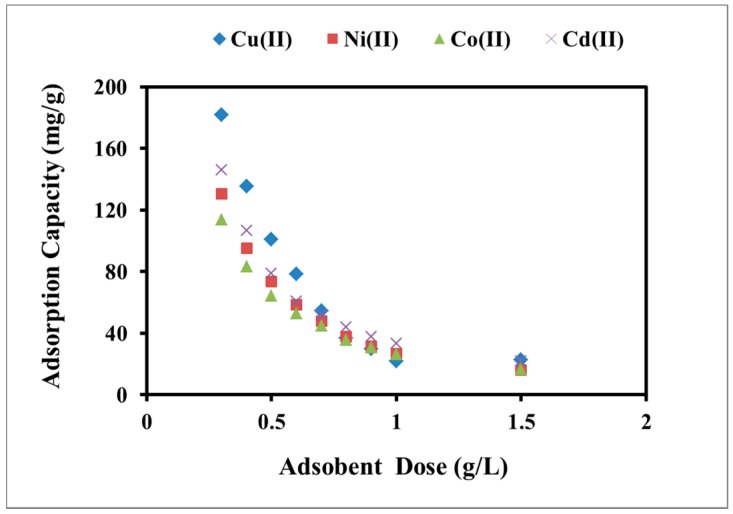
Effect of polyacrylamide functionalized Fe_3_O_4_ nanoparticles dose on the adsorption capacity of heavy metal cations at pH 6, contact time of 90 min, 140 mg/L of metal, and 25 °C.

To identify the proper adsorbent dose to reach optimum adsorption process, various amounts of polyacrylamide functionalized Fe_3_O_4_ nanoparticles were mixed with heavy metal solutions; the results are presented in Figure 7. The adsorption capacity of Cu(II), Ni(II), Co(II), and Cd(II) decreased from 181.9, 130.5, 113.8, and 145.8 mg/g to 22.9, 16.1, 16.7, and 22.0 mg/g, respectively, upon elevating the adsorbent concentration from 0.3 to 1.5 g/L for an initial metal concentration of 140 mg/L at pH 6. Such behavior is expected since at a low adsorbent dosage it will be saturated with heavy metal species and the adsorption capacity reaches its maximum value. However, when increasing the dosage gradually, there will be many active adsorption sites that will not interact with the adsorbate species. Hence, there will be a reduction in the calculated adsorption capacity under these conditions.

#### 2.2.4. Isotherms Study

**Figure 8:**
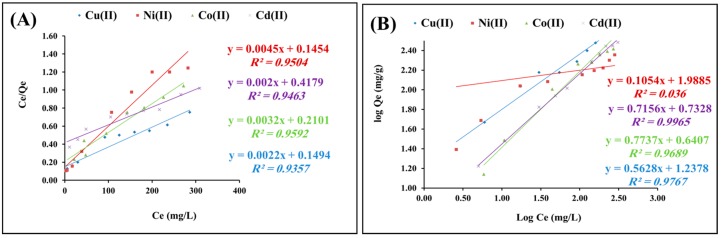
(**A**) Langmuir and (**B**) Freundlich isotherms for adsorption of heavy metal cations onto polyacrylamide functionalized Fe_3_O_4_ nanoparticles.

Studying the adsorption isotherms is crucial to identifying the distribution process of adsorbate molecules within the liquid and solid phases at the equilibrium state of adsorption process [40]. Figure 8A,B shows the adsorption isotherms for heavy metal onto polyacrylamide functionalized Fe_3_O_4_ nanoparticles at 25 °C and pH 6. The Langmuir equation (Equation (3)) was applied to the equilibrium adsorption data for heavy metal cations onto polyacrylamide functionalized Fe_3_O_4_ nanoparticles [41]:

*C_e_*/*Q_e_* = 1/(*q_max_.b*) + *C_e_*/*q_max_* (3)

where *C_e_* refers to the equilibrium concentration of the adsorbate (mg/L), *Q_e_* refers to the amount of metal ion adsorbed (mg/g), and *q_max_* and *b* refer to Langmuir constants, which are related to the maximum adsorption capacity (mg/g) and the adsorption energy, respectively. The Langmuir equilibrium constant, *K_L_*, can be obtained from Equation (4):

*K_L_* = *q_max_.b* (4)

The linear form of the Langmuir isotherm is shown in Figure 8A. The correlation coefficient, *R*^2^, for the adsorption of heavy metal onto polyacrylamide functionalized Fe_3_O_4_ nanoparticles, is 0.0.92, 0.95, 0.97, and 0.91, respectively, indicating that the adsorption parameters were well fitted by the Langmuir isotherm. These results suggest that the maximum adsorption capacity corresponds to saturated monolayer coverage of heavy metal onto polyacrylamide functionalized Fe_3_O_4_ nanoparticles, which means that the energy of the adsorption process is constant, with a low possibility of transmigration.

Contrary to the Langmuir isotherm, which assumes the formation of a monolayer of adsorbate onto the absorbent, the Freundlich isotherm postulates the formation of multiple layers of adsorbate molecules onto the surface of adsorbent material. The obtained data for adsorption of heavy metal onto polyacrylamide functionalized Fe_3_O_4_ nanoparticles were investigated using the Freundlich equation (Equation (5)):

log*q_e_* = log*K_f_* + 1/*n* log*C_e_* (5)

where *C_e_* refers to the equilibrium concentration (mg/L) and *q_e_* refers to the amount of metal adsorbed (mg/g) at equilibrium. The quantities *K_F_* and *n* refer to the Freundlich constants, with *K_F_* (mg/g) referring to the adsorbent capacity and *n* referring to the favorable nature of the process. Plots of log *q_e_* versus log *C_e_* give the slope and intercept of the line obtained corresponding to *1/n* and log*K_F_*, respectively (Figure 8B).

The calculated results of the Langmuir and Freundlich isotherm constants are given in Table 2. The adsorption of heavy metal onto polyacrylamide modified Fe_3_O_4_ nanoparticles showed good correlation with the Langmuir and Freundlich equations (*R*^2^ > 0.9) for the range of concentrations used in this study, which could suggest that the adsorption process of heavy metal onto polyacrylamide modified Fe_3_O_4_ nanoparticles involved both mono- and multilayer behavior. 

#### 2.2.5. Thermodynamic Studies

The Gibbs free energy (Δ*G*°), enthalpy (Δ*H*°), and entropy (Δ*S*°) of the heavy metal uptake onto polyacrylamide functionalized Fe_3_O_4_ nanoparticle are evaluated to explain the sorption process. They are calculated using Equations (6) and (7):

log*K_d_* = Δ*S*°/2.303*R* − Δ*H*°/2.303*RT* (6)

**Figure 9:**
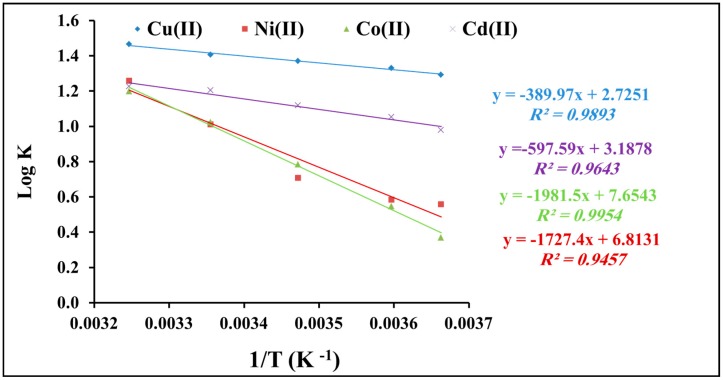
Thermodynamic factors for the adsorption of heavy metal cations onto polyacrylamide functionalized Fe_3_O_4_ nanoparticles.

Δ*G*° = − *RT* ln*K_d_* (7)

where *K_d_* refers to the equilibrium partition constant calculated based on the ratio between sorption capacity (*q_e_*) and equilibrium concentration (*C_e_*) for heavy metal uptake onto polyacrylamide-functionalized Fe_3_O_4_ nanoparticles at pH 6, *R* refers to the gas constant (8.314 J/mol K), and *T* refers to the temperature in Kelvin (K). From Equation (9), by plotting log*K_d_* vs. 1/*T* (Figure 9), Δ*H*° and Δ*S*° values can be obtained.

The values of Gibbs free energy (Δ*G*°), enthalpy (Δ*H*°), and entropy (Δ*S*°) are shown in Table 3. Δ*G*° was in the range (−6.8 to −8.6 kJ/mol) for Cu(II), (−2.9 to −7.4 kJ/mol) for Ni(II), (−1.9 to −7.1 kJ/mol) for Co(II), and (−5.1 to −7.2 kJ/mol) for Cd(II). Δ*H*° and Δ*S*° values were in the range of 52.2 to 146.6 kJ·mol^−1^ and 7.5 to 37.9 J·mol^−1^·K^−1^, respectively.

These values suggest the following characteristics: (i) the adsorption of heavy metal onto polyacrylamide functionalized Fe_3_O_4_ nanoparticles was spontaneous; (ii) the adsorption process is a typical physical process with an endothermic nature; and (iii) the process is accompanied by an increase in the degree of freedom of the adsorbed species [42,43].

#### 2.2.6. Quality Control Assessment

In order to investigate the repeatability of the results and the quality control assessment, an experiment composed of three replicate treatments for the effect of the heavy metal concentration on the adsorption capacity was conducted. Results are presented in Table 4. The results show high repeatability of the adsorption capacity for the treatment process for the tested metal ions in the concentration range of 50 mg/L to 250 mg/L. At the same time, the blank experiments that were conducted simultaneously did not show any removal behavior for heavy metal cations, indicating the effectiveness of the prepared absorbent.

## 3. Experimental Section

### 3.1. Synthesis and Characterization of Fe_3_O_4_ Nanoparticles and Polyacrylamide Functionalized Fe_3_O_4_

Chemicals including ferric chloride hexahydrate (FeCl_3_·6H_2_O), sodium acetate, sodium citrate, polyacrylamide, and glucose were obtained from Sigma-Aldrich (St. Louis, MO, USA). For synthesis of Fe_3_O_4_ nanoparticles, a precise amount of sodium acetate and FeCl_3_.6H_2_O were added to a specific volume of ethylene glycol. The mixture solution was stirred until homogeneity was achieved. Then the mixture solution was charged in a Teflon-coated stainless-steel autoclave and heated to 180 °C for 12 h. Then the autoclave was left to cool down to room temperature. The produced nanoparticles were washed with ethanol and de-ionized water for three times and then redispersed in de-ionized water.

One-step carbon coating and polyacrylamide surface functionalization of magnetic Fe_3_O_4_ nanoparticles were executed by means of a hydrothermal reaction. A known weight (0.05 g) of the previously synthesized Fe_3_O_4_ nanoparticles was dispersed in a known volume of 0.5 M glucose solution and thereafter a specific amount of polyacrylamide (0.5 g) was introduced to the mixture solution. The mixture was loaded in a Teflon-coated stainless-steel autoclave and heated at 200 °C for 3 h. The produced carbon-coated and polyacrylamide functionalized Fe_3_O_4_ nanoparticles were washed with ethanol and deionized water three times [15]. However, for ease, the produced material will be described as “polyacrylamide functionalized Fe_3_O_4_”. A scanning electron microscope (JSM-7600F, JEOL, Tokyo, Japan) was utilized to obtain the sample morphology. Transmission electron microscopy (TEM) characterization was conducted to study the carbon coating and dispersity of magnetic Fe_3_O_4_ nanoparticles using a JEM-2100F electron microscope, Tokyo, Japan. Powder X-ray diffraction (XRD) measurements were conducted using X’Pert PRO MPD, PANalytical, Almelo, The Netherlands) on bare and carbon-coated polyacrylamide functionalized samples to investigate the crystalline character of the samples. To elucidate the functionality after carbon coating and the functionalization step, Fourier transform infrared (FTIR) spectra were recorded using a Vertex-80 spectrometer (Bruker, Billerica, MA, USA).

### 3.2. Adsorptive Removal of Heavy Metal Ions on Polyacrylamide Functionalized Fe_3_O_4_

Batch procedures were used to study the adsorption of heavy metal cations on polyacrylamide functionalized Fe_3_O_4_ in term of kinetics and thermodynamics, as described in Wang et al. [44]. Stalk metals solution (300 mg/L) were prepared from nitrate salts, which were bought from Sigma-Aldrich. The other metal concentrations (100 mg/L and 200 mg/L) were prepared by dilution. Ten milliliters of multi-metal cation Cu(II), Ni(II), Co(II), and Cd(II) solution were mixed with 0.01 g of polyacrylamide modified Fe_3_O_4_ in Erlenmeyer flasks. The mixture was subjected to shaking at a speed of 150 rpm for a desired time. By the completion of the adsorption test, the adsorbent was separated from the solution by the assistance of an external magnetic field. For quality control, blank samples without polyacrylamide functionalized Fe_3_O_4_ adsorbent were run simultaneously in all experiments. In addition, triplicate experiments were completed to ensure the repeatability of the adsorption capacity values [45]. The determinations of the adsorptive removal capacity were done by measuring the metal ion concentrations with atomic absorption spectroscopy (AAS) before and after the adsorption process, and then applying the following equation:

*q_e_* = (*C*_0_ − *Ce*). V/M (8)

where *q_e_* refers to the adsorption capacity (mg/g), *C*_0_ refers to the initial concentration of metal ion solution, *C_e_* refers to the equilibrium concentration of metal ion solution, V refers to the volume of the metal ion solution, and M refers to the mass of the adsorbent (g).

## 4. Conclusions

In this work, a one-step synthesis process was implemented simultaneously for developing a carbon coating layer together with imparting polyacrylamide functionalization for Fe_3_O_4_ nanoparticles using the hydrothermal technique. TEM observation indicates that the carbon coating matrix surrounding the magnetic Fe_3_O_4_ nanoparticles was homogenously distributed within the matrix. FTIR spectra indicated the presence of amide I and II groups, which suggests the polyacrylamide functionalization for carbon coated Fe_3_O_4_. The polyacrylamide functionalized Fe_3_O_4_ nanoparticles produced showed resistance to leaching in an acidic medium due to protection by the carbon layer. The ability of the Fe_3_O_4_ nanoparticles to adsorb heavy metals was improved through functionalization with a polyacrylamide layer. As the adsorptive removal process parameters were optimized, the pseudo-second-order kinetic model was more suitable to describe the process. The adsorption data were well fitted by Langmuir and Freundlich isotherms. The thermodynamic parameters, including Gibbs free energy (Δ*G*°), enthalpy (Δ*H*°), and entropy (Δ*S*°), were investigated, and the results suggested spontaneous behavior for the heavy metal cations adsorption using polyacrylamide functionalized Fe_3_O_4_ nanoparticles. Hence, the prepared adsorbent is recommended for separation and removal of heavy metals from wastewater by the adsorption process.

## Figures and Tables

**Table 1:** Kinetic constant parameters obtained for adsorption of heavy metal onto polyacrylamide functionalized Fe_3_O_4_ nanoparticles.

		Pseudo-First-Order			Pseudo-Second-Order		
	*q_e_*, exp (mg/g)	K_1_ (min^−1^)	*q_e_*, cal (mg/g)	*R*^2^	*k*_2_ (g/mg·min)	*q_e_*, cal (mg/g)	*R*^2^
Cu(II)	194	0.031	239.8	0.91	5.7 × 10 ^−4^	200.18	0.98
Ni(II)	144.3	0.030	128.8	0.92	6.8 × 10^−4^	150.7	0.99
Co(II)	128	0.045	208.9	0.87	12.8 × 10^−4^	130.6	0.98
Cd(II)	161	0.035	114.8	0.93	6.8 × 10^−4^	167.7	0.99

**Table 2:** Langmuir and Freundlich constants for the adsorption of Cu(II), Ni(II), Co(II), and Cd(II) onto polyacrylamide functionalized Fe_3_O_4_ nanoparticles.

	Langmuir Constants				Freundlich Constants		
	K_L_	b	Q_max._	*R*^2^	K_F_	*n*	*R*^2^
Cu(II)	5.9	0.013	454.5	0.92	17.4	1.78	0.97
Ni(II)	6.9	0.031	222.2	0.95	22.6	2.42	0.92
Co(II)	4.7	0.015	312.5	0.97	5.09	1.38	0.96
Cd(II)	2.3	0.0043	526.3	0.91	5.42	1.39	0.99

**Table 3:** Thermodynamic parameters for adsorption of heavy metal cations onto polyacrylamide functionalized Fe_3_O_4_ nanoparticles.

	Temperature T(K)	Thermodynamic Parameters		
		Δ*G*° (kJ/mol)	Δ*S*° (J/mol/K)	Δ*H*° (kJ/mol)
Cu(II)	273	−6.8	52.2	7.5
	278	−7.1		
	288	−7.6		
	298	−8.0		
	308	−8.6		
Ni(II)	273	−2.9	130.5	33.1
	278	−3.1		
	288	−3.9		
	298	−5.8		
	308	−7.4		
Co(II)	273	−1.9	146.6	37.9
	278	−2.9		
	288	−4.3		
	298	−5.8		
	308	−7.1		
Cd(II)	273	−5.1	61.0	11.4
	278	−5.6		
	288	−6.2		
	298	−6.9		
	308	−7.2		

**Table 4:** Impact of heavy metal concentration on the adsorption capacity (*n*=3).

Metal ion Concentration (ppm)	Adsorption Capacity (*Q_e_* ± SD)			
	Cu	Ni	Co	Cd
50	105.1 ± 4.5	105.3 ± 3.5	88.1 ± 5.5	74.4 ± 6.9
100	156.7 ± 6.7	132.2 ± 1.9	180.9 ± 7.2	104.4 ± 5.1
150	192.2 ± 1.9	144.1 ± 2.3	184.4 ± 1.9	156.7 ± 6.7
200	250.0 ± 3.3	153.3 ± 5.8	186.7 ± 5.8	187.8 ± 5.1
250	301.7 ± 2.4	170.0 ± 4.7	230.0 ± 2.4	231.7 ± 2.4

## Supplementary Materials

Supplementary materials are available online.
