# Supplementary material for: One-Step Carbon Coating and Polyacrylamide Functionalization of Fe3O4 Nanoparticles for Enhancing Magnetic Adsorptive-Remediation of Heavy Metals
Source: Molecules. 2017 Nov 27;22(12):2074. doi: 10.3390/molecules22122074 (PMC6149930; doi:10.3390/molecules22122074)
Supplement: Supplementary file 1 [file molecules-22-02074-s001.pdf]

One-Step Carbon Coating and Polyacrylamide Functionalization of Fe_3O_4 Nanoparticles for Enhancing Magnetic Adsorptive-Remediation of Heavy Metals

Acid Stability Test

First, 0.1 g Fe_3O_4 and polyacrylamide-functionalized Fe_3O_4 were put separately in 50-mL tubes and 10 mL of 0.1M HCl was added. The mixtures were shaken for 6 h. The Fe_3O_4 and polyacrylamide-functionalized Fe_3O_4 were separated from the acid solution by the use of a magnet. The dissolved Fe ions were determined by AAS. The concentration of Fe ions in the case of Fe_3O_4 was considered to be 100% dissolution.

For calculation of the dissolution %:

Dissolution %= (concentration of Fe ions in case of polyacrylamide-functionalized Fe_3O_4 /concentration of Fe ions in case of Fe_3O_4)*100.

Protection % was calculated as follows:

Protection% = (1-(concentration of Fe ions in case of poly-acrylamide modified Fe3O4 /concentration of Fe ions in case of Fe_3O_4)*100.

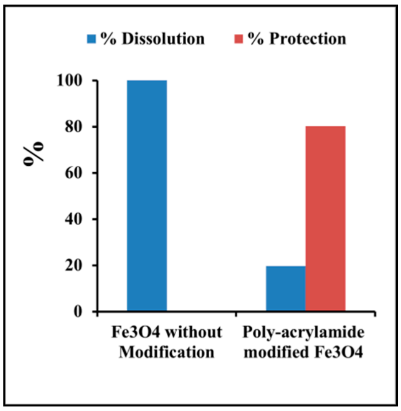

Figure S1. Determination of acid stability of Fe_3O_4 and polyacrylamide-functionalized Fe_3O_4.

To evaluate the gained stability for the Fe_3O_4 after functionalization with polyacrylamide, the effect of 0.1 M HCL on the Fe_3O_4 and polyacrylamide-functionalized

Fe_3O_4 was investigated. Figure S1 shows that the dissolution percentage in the case of unfunctionalized Fe_3O_4 was high (considered 100%) compared with 20% in the case of polyacrylamide-functionalized Fe_3O_4. This means that there is a protection from dissolution in an acidic medium, making the polyacrylamide-functionalized Fe_3O_4 more suitable for heavy metals adsorption, which usually occurs in an acidic medium.
